# A Killer–Rescue system for self-limiting gene drive of anti-pathogen constructs

**DOI:** 10.1098/rspb.2008.0846

**Published:** 2008-09-02

**Authors:** Fred Gould, Yunxin Huang, Mathieu Legros, Alun L Lloyd

**Affiliations:** 1 Department of Entomology, North Carolina State UniversityRaleigh, NC 27695, USA; 2 Department of Mathematics and Biomathematics Graduate Program, North Carolina State UniversityRaleigh, NC 27695, USA

**Keywords:** gene drive, selfish genes, *Aedes*, *Anopheles*, dengue, malaria

## Abstract

A number of genetic mechanisms have been suggested for driving anti-pathogen genes into natural populations. Each of these mechanisms requires complex genetic engineering, and most are theoretically expected to permanently spread throughout the target species' geographical range. In the near term, risk issues and technical limits of molecular methods could delay the development and use of these mechanisms. We propose a gene-drive mechanism that can be self-limiting over time and space, and is simpler to build. This mechanism involves one gene that codes for toxicity (killer) and a second that confers immunity to the toxic effects (rescue). We use population-genetic models to explore cases with one or two independent insertions of the killer gene and one insertion of the rescue gene. We vary the dominance and penetrance of gene action, as well as the magnitude of fitness costs. Even with the fitness costs of 10 per cent for each gene, the proportion of mosquitoes expected to transmit the pathogen decreases below 5 per cent for over 40 generations after one 2 : 1 release (engineered : wild) or after four 1 : 2 releases. Both the killer and rescue genes will be lost from the population over time, if the rescue construct has any associated fitness cost. Molecular approaches for constructing strains are discussed.

## 1. Introduction

Genetic alteration of insects to decrease transmission of human pathogens such as dengue virus and *Plasmodium* is an appealing concept (Curtis 1968; Gould & Schliekelman 2004), and is now on the verge of empirical feasibility (James 2005; Chen *et al*. 2007). Engineered mosquito strains with transgenes that decrease transmission of one serotype of dengue virus and one species of malaria-causing *Plasmodium* have been developed in the laboratory (Ito *et al*. 2002; Franz *et al*. 2006). Simply releasing such non-vectoring strains of mosquitoes into the wild will not curtail dengue or malaria transmission unless the genes coding for refractoriness can spread and increase in the natural environment. That can only happen if the pathogen-refractory genes confer higher fitness to the individuals bearing them, if they are linked to other genes that increase fitness, or if they are linked to ‘selfish’ genes that have an ability to increase in frequency through super-Mendelian inheritance or by negative epistatic genetic interactions (Sinkins & Gould 2006).

A large number of evolutionarily and mechanistically diverse selfish genetic elements exist in natural populations of plants, animals and microbes (Burt & Trivers 2006). Among the varied properties of these selfish elements are the rate at which each one can increase in frequency and the threshold frequency below which they are lost from a population. Molecular geneticists have attempted to harness selfish genetic elements with high rates of increase and low thresholds (James 2005). Under ideal conditions, such elements could spread throughout a species based on the release of a single fertile female. Many early attempts to use transposons, one element with these properties, as a gene driver in mosquitoes failed (O'Brochta *et al*. 2003; Sethuraman *et al*. 2007), but recent success with building an artificial selfish *Medea* element in *Drosophila* has provided hope that a similar artificial element could be incorporated into mosquitoes (Chen *et al*. 2007; Fischetti 2008). Based on population-genetic theory and laboratory experiments, natural and artificial *Medea* elements are expected to spread within and among wild populations from relatively low initial frequencies to fixation or near fixation (Wade & Beeman 1994).

If a synthetic *Medea* element can be developed to function in the mosquito species *Aedes aegypti* or *Anopheles gambiae*, which vector dengue and malaria, respectively, it would seem to be an ideal candidate for spreading anti-pathogen genes. However, one problem with *Medea* is that it might spread too fast, and too far geographically, before all of the potentially affected communities and countries were agreed that such spread was beneficial to each of them (Scott *et al*. 2002; Knols *et al*. 2006). Furthermore, its broad spread to high frequency could select for rapid pathogen adaptation. In the step-by-step approach developed for environmental release of other genetically engineered organisms (e.g. NRC 2000, 2002), there would be many laboratory and field experiments to conduct before the release of an organism with an active *Medea* element, or any other genetic element that was expected to spread widely. Some researchers (e.g. Benedict & Robinson 2003) have called for the release of genetically engineered insects that are sterile, or in other ways self-limiting before any release of insects with *Medea*-like properties.

In this paper, we propose and theoretically evaluate a novel, self-limiting gene-drive mechanism that has not been previously considered. The properties of this gene-drive mechanism derive from epistatic interactions that occur between a gene that we will call the ‘killer’ (autocidal) gene and a gene that we will call the ‘rescue’ gene, when these two genes are inserted at independently segregating loci. The killer gene could code for a toxic protein or RNA, and the rescue gene could code for an enzyme or an RNA that neutralizes the impact of the killer gene. Any individual that receives one or more copies of the killer gene from its parents but no copies of the rescue gene would die. If the transgenic construct with the rescue gene was also engineered to include an anti-pathogen gene, the frequency of the anti-pathogen gene in a population would always be exactly the same as the frequency of the rescue gene.

This system has the benefit of being less complex at the molecular level than other gene-drive mechanisms, so its construction in non-model organisms is likely to be more feasible with currently available tools. The killer–rescue system is predicted to locally spread an anti-pathogen gene to high frequency for only a limited period of time, so it presents less of a risk than other gene-drive systems. The self-limiting properties of this system derive from fitness costs associated with the transgenic constructs as well as general population-genetic properties of the interaction between killer and rescue genes. In most discussions of gene drive (e.g. Sinkins & Gould 2006), emphasis is placed on trying to build engineered constructs with low or no fitness costs. For some applications of this killer–rescue system where the goal is a short-term test of an anti-pathogen gene, it might not be necessary or even useful to aim for low fitness costs.

Here, we present simple two- and three-locus population-genetic models that describe the properties of this system when one rescue gene and one or two copies of a killer gene are each engineered into independently assorting loci of the transgenic strain. We also discuss some potential genes and genetic tools that could be used to build the system.

## 2. Model development and rationale

In the simplest form of our model, it is assumed that one copy of the killer gene is sufficient to kill any individual that does not have the rescue gene. It is also assumed that carrying one copy of the rescue gene is sufficient to completely suppress the action of the killer gene, no matter how many copies of the killer gene are present. These assumptions are based on the idea that the killing is caused by the expression of a gene that produces a toxic protein or RNA, and that the rescue is caused by a gene that renders individuals immune to the action of the killer alleles, no matter what the ratio is of the killer to rescue alleles. It is assumed that one copy of the anti-pathogen gene completely interferes with pathogen transmission. In the simple form of the model, it is always assumed that homozygous engineered individuals are released at a 1 : 1 sex ratio.

Throughout this paper, we refer to the killer allele as ‘K’ and the rescue allele as ‘R’. The alternate null alleles are referred to as ‘k’ and ‘r’, respectively. When there are two insertions of the killer alleles, the second insertion will be referred to as K′. Each locus at which a K or R allele is inserted has independent recombination with all other loci with insertions. The model is completely deterministic, and it assumes infinite population size and random mating. In §3, we consider an allele to be lost from the population if it is below a frequency of 0.001 and decreasing.

If there are no fitness costs associated with carrying either R or K (when it is suppressed), then for the two-locus case the fitness of seven of the nine genotypes is simply 1.0 (KKRR, KKRr, KkRR, KkRr, kkRR, kkRr and kkrr). By contrast, the fitness of genotypes Kkrr and KKrr is 0 because they have one or two copies of the K allele and no R alleles.

The fitness costs associated with being homozygous for the killer and rescue genes are *c*_K2_ and *c*_R2_, respectively. We assume that the fitness costs are additive both at a locus and between loci, i.e. the costs to heterozygotes, *c*_K1_ and *c*_R1_, are half of the homozygote costs, and, when an individual has both the killer and rescue alleles, the total cost is the sum of the costs due to the killer and rescue alleles. The iterative equations that describe the dynamics of this simple model with one K and one R insertion are given in the electronic supplementary material, §IV.

The simple model is modified by adding female-specific and partial killing. Other modifications to the model including variation in the dominance of fitness costs, and male-only releases, are presented and discussed in the electronic supplementary material. The C++ computer code is available from the authors.

## 3. Model exploration

Detailed mathematical analysis of the dynamics, equilibria and introduction thresholds are provided in the electronic supplementary material, §§V, VI and VII. Here, we present time-series dynamics for a set of potentially realistic cases.

### (a) No fitness costs

Figure 1 depicts the temporal dynamics of the simplest model, with no fitness costs, over a biologically relevant period of 120 generations (equivalent of approx. 10 years for *A. aegypti* in some tropical climates). Figure 1*a* shows the results of a single release of a homozygous engineered strain with one K insertion and one R insertion, and a 1 : 1 sex ratio. The release ratio is two engineered insects for each native insect, so the initial frequency of homozygous engineered insects is approximately 0.66. As shown in figure 1*a*, the frequency of K drops slightly over the 120 generations, while the frequency of R increases to approximately 0.99. The frequency of insects within the population that can transmit the pathogen is equal to the frequency of kkrr (i.e. the wild-type) because only rr individuals can transmit the pathogen, and any individual that is homozygous for rr but carries a K allele (KKrr or Kkrr) will die.

The result of a similar release with one-quarter the number of engineered insects (one released insect per two native insects, resulting in an initial frequency of approx. 0.33 engineered insects) is shown in figure 1*b*. Here, the K allele is nearly lost from the population while the R allele increases to 0.86 due to mortality of rr genotypes caused by the K allele. If the small release is repeated four times over the first four generations (in a population that maintains a stable density) so that the total number of released insects is equal to the number released in a single-generation release depicted in figure 1*a*, then the frequency of K after 120 generations is substantially higher, as can be seen by comparing figure 1*a*,*c*.

Figure 1*d* depicts the dynamics of a single 1 : 2 release of a homozygous strain that has two independently assorting loci with insertions of the same killer gene (K and K′) and one locus with a rescue gene insertion. Even though the release frequency is the same as in figure 1*b*, the frequency of R after 120 generations is substantially higher, and the frequency of individuals that can transmit the pathogen is less than 1 in 1000. Results in figure 1 do not address the issue of space. If the engineered insects were released in a local area, native immigrants into the local area would reduce the frequency of the R allele.

The solid lines at the top of figure 1*a–d* depict the relative fitness of the populations after the release of the killer–rescue strain. In all cases, the fitness of the population decreases somewhat in the generations immediately after the release due to the impact of the killer allele, but over time, the relative fitness approaches 1.0 as either the killer allele is lost from the population or the rescue allele approaches fixation.

It would be impractical to present individual time-series figures for all possible release ratios. Figure 2 summarizes the long-term dynamics that are predicted following single releases of a homozygous strain with one insertion each of K and R for a range of initial release frequencies. The frequencies of the K and R alleles in successive generations are plotted as dots on the figure, with lines joining the dots to aid visualization of the trajectories that result from different releases. Because the introduction occurs via homozygous individuals, the initial allele frequencies lie on the solid black diagonal line. (Black arrows indicate the points on this line that represent the 2 : 1 and 1 : 2 releases that are detailed in figure 1*a*,*b*.)

In the long term, each trajectory approaches an equilibrium. Depending on the release fraction, this equilibrium will either include (green line) or not include (red line) any K alleles. In all cases where some K alleles are present at equilibrium, the R allele is fixed, and no individuals can transmit the pathogen. The trajectory that separates these two outcomes is close to (although not exactly) a straight line, which allows an approximate value of the release threshold that delineates these two qualitatively different outcomes to be calculated. Details of this calculation appear in the electronic supplementary material, §VII, and give the release threshold as approximately 0.354. Numerical simulation can determine the precise value of the threshold and we find it to lie between 0.350 and 0.351. (An alternative visualization, in which the frequencies of three of the four possible gamete types, KR, kR and kr, are plotted, is presented in the electronic supplementary material, §V, together with a mathematical analysis of all possible equilibria.)

### (b) Equal additive fitness costs for K and R alleles

In figures 1 and 2, it is assumed that the individuals with one or two R alleles always have a relative fitness of 1.0, and those with one or two K alleles have a fitness of 1.0 when R is present. This assumption is now relaxed, first by adding identical fitness costs to the R and K alleles. We assume here that fitness costs are additive, so *c*_K1_=0.5 *c*_K2_. Similarly, *c*_R1_=0.5 *c*_R2_. (We explore the model with other assumptions about dominance inheritance of the fitness costs in the electronic supplementary material, §I.) Results of the model when *c*_K2_ and *c*_R2_ are each equal to 0.1 are shown in figure 3*a–d*, where the types of releases are identical to those in figure 1*a–d*. With this 0.1 fitness cost, the K and R alleles are lost (or are at extremely low frequency) by generation 120 if there is a 1 : 2 release (figure 3*b*,*d*), even when there are two insertions of K. When there is a single 2 : 1 release or four 1 : 2 releases (figure 3*a*,*c*), the frequency of pathogen transmitting individuals is reduced below 5 per cent for 50 and 70 generations, respectively. However, in both cases, K is lost before generation 120 and R is less than 0.30 by generation 120.

Even if *c*_K2_ and *c*_R2_ are set lower, at 0.05, and the release is large enough to result in an initial frequency of 0.95 of R and K (19 : 1 release), and there are two insertions of K, the R alleles are lost by generation 600 (not shown). This loss of R occurs because both Ks are almost lost by generation 300 due to selection and fitness costs.

To further understand the selection factors that impact the pattern of allele frequency changes, we have plotted the fitness of each allele over time in the electronic supplementary material, §III. A mathematical analysis of possible equilibria under conditions where both K and R have fitness costs is given in the electronic supplementary material, §V.

### (c) Additive fitness costs only for K or only for R

The fitness costs associated with the K and R constructs could be very different. Both could have costs associated with the insertion site, but costs to the K construct could also come from low levels of toxin production even when the R allele is homozygous. A fitness cost to the R construct could occur if the expression of the rescue substance (protein or RNA) had effects other than interfering with the action of the construct K. Because the rescue gene and the anti-pathogen gene are part of the same construct, any negative effects of the anti-pathogen gene on the insect's fitness would be part of the fitness cost associated with the R allele.

It is difficult to predict just what the fitness cost differences would be between R and K, but to provide insight into how such differences would affect the dynamics of the gene-drive system we compared the cases above (figure 3*a–d*), where both *c*_K2_ and *c*_R2_ were 0.10 to the extreme case where either *c*_K2_ or *c*_R2_ was set at 0.20 while the other cost was set at 0.

Results shown in figure 4 demonstrate that the fitness effects from the K allele have different impacts on allele frequencies than the fitness costs associated with the R allele. If the 0.20 fitness cost is associated with the R construct and there is no cost to K, then when there is a single 2 : 1 release, the frequency of R begins to drop substantially before generation 120 (figure 4*a*). By contrast, the R allele frequency reaches a high equilibrium level when the 0.20 fitness cost is associated with K and there is no fitness cost for R (figure 4*b*). If four 1 : 2 releases are conducted and the 0.20 cost is associated with K, the results (figure 4*d*) are generally similar to those with the single 2 : 1 release. However, when the 0.20 cost is associated with R, the four small releases result in a more sustained impact on pathogen transmission (figure 4*c*) than the single larger release (figure 4*a*). This occurs because in the single-generation intervals between each of the four releases, K and R are increasing due to selection, and the final frequencies of K and R after the four releases are therefore higher than those after the single 2 : 1 release. This result contrasts with the behaviour of engineered underdominance constructs where fewer total insects need to be released in a single release than in multiple releases (Magori & Gould 2006).

When the fitness costs associated with the R insertion are large, it is not surprising to find that the R allele is eventually lost. When there is no fitness cost associated with K, and *c*_R2_ is only 0.05 (resulting in heterozygotes with a 0.975 relative fitness), the R allele can remain in the population for over 1000 generations if the release ratio is high. However, both the K and R alleles are lost by generation 1000 if the release results in an initial frequency below approximately 0.50. Thus, even with no immigrants from beyond the spatial range of the release, the killer–rescue system can be self-limiting over time with fitness costs that are typically too small to be detected experimentally.

All potential equilibria when only R or only K has associated fitness costs are explored in the electronic supplementary material, §V.

### (d) Partial killing and additive inheritance of K or R

In all of the results that follow we use a 2 : 1 release and assume that *c*_K2_=*c*_R2_=0.10 and the cost is additive.

Partial killing can be viewed as killing less than 100 per cent of rr individuals when K is homozygous. We compare the two cases: (i) 50 per cent of males and females are killed and (ii) 0 per cent of males and 100 per cent of females are killed. When the killing by K is dominant, results are identical if only rr females die or 50 per cent of all rr individuals die (figure 5*a*). Under both of these conditions, pathogen transmission is not reduced as much as when there is a similar release with 100 per cent killing (shown in figure 3*a*).

If all KKrr individuals die, but only 50 per cent of Kkrr individuals die, then killing is considered additive. It is interesting that under this condition the results of the model are identical to those in which there is complete dominance but only 50 per cent mortality (the trajectories of allele frequencies in figure 5*a* therefore also represent this case).

If the killing impact of K is completely dominant and rescuing by R is additive, there is a very different result, with R approaching fixation (figure 5*b*) for almost 200 generations (not shown). This result seems counter-intuitive, but it makes sense because at frequencies above 0.90 the additive genetic variance for fitness associated with R is much greater when R is additive than when it is dominant in effect. This could be important to consider when building the rescue constructs.

## 4. Discussion

A decrease in the incidence of malaria or dengue fever is expected to result in dramatic benefits to society; so any mechanism for accomplishing this, including the use of genetically engineered mosquitoes, must be considered. Although strong anti-dengue and anti-*Plasmodium* transgenes, as well as strong gene drivers, may be engineered into mosquitoes within the next 5–10 years, it is not expected that the first field releases of transgenic mosquito strains will carry a strong gene driver that could presumably spread throughout a species' range based on the release of a small number of individuals (Gould 2008). Indeed, such a first release would be problematic from both a societal and scientific perspective. Perhaps the worst outcome would be if a strong gene-drive mechanism spreads an anti-pathogen gene to high frequency over a wide area and then the pathogen rapidly adapted to the gene product.

A cautious approach to the use of engineered refractory mosquito strains would have a number of steps built into a release strategy that would ensure that any final releases using strong gene-drive systems would have a high likelihood of success. If the recent USDA-APHIS permitting process for the release of transgenic pink bollworm becomes a precedent for future releases of transgenic insects, then methodical small steps towards the release of insects with strong drive mechanisms are expected (but see Alphey *et al*. 2002). The first releases of transgenic mosquitoes might be strains that simply carry a marker gene such as green fluorescent protein. Further on, transgenic mosquitoes that are refractory to a pathogen but without any drive mechanism might be released and monitored over time (Alphey *et al*. 2002).

Our contention is that at some time between the release of a mosquito strain with a non-driven refractory gene and the release of a strain with a strong drive mechanism such as a *Medea* or meiotic drive construct, there will be a need to test a strain with a refractory gene and some kind of self-limiting drive mechanism. The killer–rescue system proposed and described here could serve as such an intermediate step. Our deterministic non-spatial model shows that, even when there is only a very small fitness cost associated with the rescue construct, both the R and K alleles will be lost from the population. When there are very local releases into small, spatially structured, populations of *Ae. aegypti*, these alleles are expected to be lost over time, even if there are no fitness costs (M. Legros *et al*. 2008, unpublished results).

It has generally been assumed that one goal of laboratories that are engineering mosquitoes for gene drive should be production of strains with the lowest possible fitness costs, and that lines with large fitness costs should be discarded. However, it is reasonable to expect that regulatory agencies will want the first released strains to be lost from native populations in a relatively short period of time. In anticipation of the possibility of such requests or requirements, it might be a good idea to save some lines that have substantial fitness costs due to defined mechanistic causes.

In addition to the killer–rescue genetic drive system limiting itself over time, this drive mechanism has the advantage of being relatively simple to construct compared with already-proposed gene-drive mechanisms (see Sinkins & Gould 2006 for a review), especially if some associated fitness costs are acceptable or desirable.

In its simplest form, a killer–rescue system could be composed of (i) a genetic construct with a gene coding for a miRNA or RNAi sequence that silences any single gene in the insect that is critical to survival, and (ii) a construct with a synthetic sequence of the critical gene that is not recognized by the interfering RNA fragments (see Chen *et al*. 2007). During the research phase, the killer gene would probably need to be linked to a conditional promoter such as a heat-shock promoter or a Tet-on or Tet-off promoter (Bello *et al*. 1998; Coates *et al*. 1998; Gong *et al*. 2005) to make the testing of the system efficient. However, in the final strain, the killer and rescue genes could be constitutively expressed, assuming that the strain was built by first adding the rescue construct, with the killer gene being added subsequently. In most cases, it would be useful to only express the killer and rescue genes in a specific tissue during a specific life stage in order to decrease the fitness costs to a range between 0.05 and 0.20. Broader expression of the rescue than the killer gene might be important to ensure that the killer gene was always silenced, but this would depend on the specific molecular system.

Instead of the killer gene being a sequence for the expression of miRNA or RNAi, it could actually be a gene that codes for a toxic protein. In this case, the rescue sequence would code for a product that detoxified the protein or silenced the expression of the killer gene. It is expected (but not certain) that both the systems described here would show dominant effects of the killer and rescue genes. If two copies of the rescue allele were needed to completely overcome the effects of the killer gene, then the rescue trait might be inherited in a more additive fashion that could, somewhat counter-intuitively, lead to more sustained high frequencies of the rescue gene construct (see §3*d*).

Although results from the model indicate that a gene that kills both males and females that lack the rescue construct would be more efficient than a gene that only killed females, there could be some advantages to certain female-killing genes. An anti-dengue virus RNAi construct has been tested in *Ae. aegypti* and shown to be effective (Franz *et al*. 2006). This RNAi construct is regulated by a carboxypeptidase promoter and is therefore only transcribed in the female midgut tissues after a blood meal (Moreira *et al*. 2000; Franz *et al*. 2006). A rescue gene could presumably be incorporated into that construct and would also be regulated by the carboxypeptidase promoter. If a toxin-producing gene inserted on a separate chromosome also used this blood-meal-activated promoter, it is feasible that the resulting strain would only kill females and would have low fitness costs owing to tissue/stage-specific expression. Most importantly, it would render females that had both the K and R constructs resistant to dengue virus proliferation in their midguts. Males would not need the anti-dengue gene expression since they do not bite humans. If the engineered construct were organized so that the promoter could not cause transcription of the rescue gene unless the anti-pathogen gene was also transcribed (Chen *et al*. 2007), this would decrease the chance that a rescue construct that had defective anti-pathogen gene expression could increase in the population. A similar system could presumably be developed for *Anopheles* species that transmit malaria.

As discussed above, in some cases, it would be beneficial to use a strain with a significant fitness cost to the rescue/anti-pathogen construct that would be quickly lost from the population after a single release. An additional useful attribute of a system with such fitness costs is that after the initial release, the frequency of the rescue/anti-pathogen construct could be maintained in the population, if desired, by subsequent smaller releases of mosquitoes with both constructs. Once these follow-up releases were stopped, the frequency of the rescue/anti-pathogen construct would decrease. This attribute provides both the researcher and the local community with significant control over the fate of a release.

Because it is difficult to assess the in-field fitness costs of mosquito strains in the laboratory, it would be critical to conduct large field-cage tests of the engineered strains prior to any release so that the magnitude of *c*_K2_ and *c*_R2_, and their dominance values, could be estimated. Even estimates from large field cages cannot always predict in-field fitness. Therefore, it is at least possible that a strain will have higher fitness costs in pre-release tests than those seen in the field. One property of this killer–rescue system is that when releases are done at low frequencies, the killer gene construct is always expected to be lost quickly, even if it has no fitness costs. If the rescue construct has even a small fitness cost, it too would be lost from an isolated population. If there is immigration of wild-type insects into the area where the release was conducted, the rescue construct would be diluted out of the population because there would be no killer gene to drive it. This all argues for the first field releases to involve a small number of engineered mosquitoes yielding low initial frequencies of the K and R alleles that could be monitored based on the K and R constructs that also coded for easily assayed markers such as green fluorescent protein. Allele frequency changes found through such monitoring would provide estimates of in-field fitness costs.

Here, we theoretically explored only cases with one or two killer genes and one rescue gene. Our results provide a general prediction of the usefulness of adding more killer and rescue genes that are inherited independently. It might be useful to insert killer and rescue genes at positions on the same chromosome that would lead to limited recombination. The model framework used here assumed infinite population size and random mating. There was no age structure to the populations, and there were no stochastic processes in the model. This type of simple model has been successfully used to gain insights into many population-genetic processes. However, it will be critical to further examine the dynamics of the killer–rescue system with more detailed computer simulation models that include the specific biology of the target mosquito populations (e.g. Legros *et al*. in preparation).

## Figures and Tables

**Figure 1:**
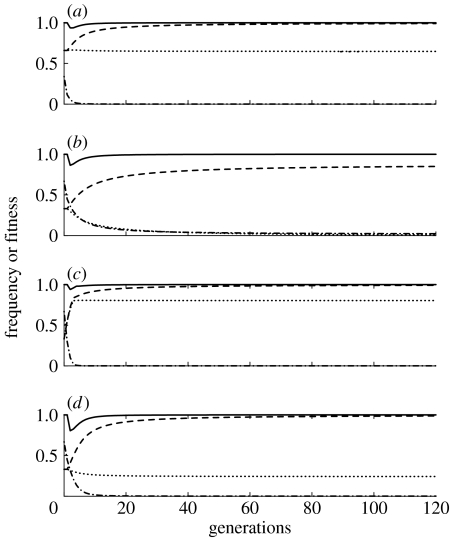
Allele frequencies, wild genotype frequency and population fitness over time after the release of killer–rescue strains with no fitness costs to the K or R alleles. (*a*) Single release of the homozygous killer–rescue strain at a 2 : 1 ratio relative to the wild population, resulting in a K and R initial frequency of 0.66, one killer gene. (*b*) As in (*a*) except that there is a 1 : 2 release, initial frequency of 0.33. (*c*) Releases at 1 : 2 ratio in each of the first four generations, one killer gene. (*d*) A single release at a 1 : 2 ratio of a strain that is homozygous for two insertions of K and one insertion of R. In all cases, the sex ratio of released individuals is 1 : 1. Dash-dotted line, wild-type (kkrr); dotted line, K allele; dashed line, R allele; solid line, mean fitness.

**Figure 2:**
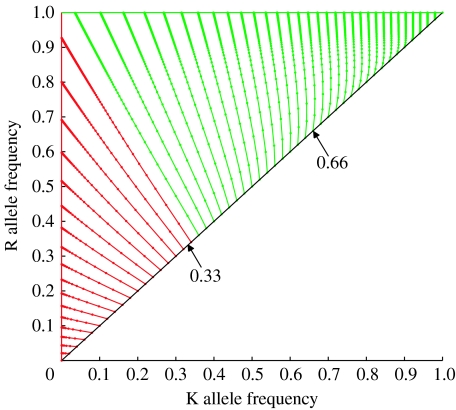
The relationship between the initial frequency of the released killer–rescue strain and the trajectory towards equilibrium of the killer and rescue allele frequencies. The fitness costs of K and R are set at zero. The allele frequencies in successive generations are depicted as dots, with the lines joining the dots to aid visualization of the trajectories. (The closer the dots are along a line, the less is the change in the gamete frequencies per generation; so, as the equilibrium is approached, the dots appear to overlap.) The R fixed line of equilibria is shown as a solid green line and the K absent line of equilibria as a solid red line. Trajectories are coloured red or green, according to which of these two outcomes occurs in the long term. Initial conditions lie on the solid black line.

**Figure 3:**
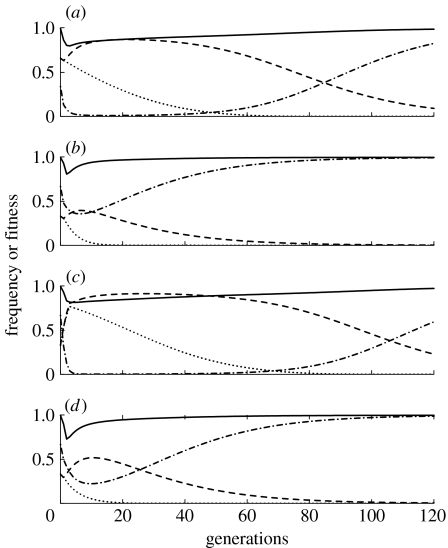
Allele frequencies, wild genotype frequency and population fitness over time, following the release of killer–rescue strains with homozygous fitness costs, *c*_K2_ and *c*_R2_, each equal to 0.1, and heterozygous fitness costs, *c*_K1_ and *c*_R1_, each equal to 0.05. (*a*) Single release of the homozygous killer–rescue strain at a 2 : 1 ratio relative to the wild population, resulting in a K and R initial frequency of 0.66. (*b*) As in (*a*) except that there is a 1 : 2 release, initial frequency of 0.33. (*c*) Releases at 1 : 2 ratio in each of the first four generations, one killer gene. (*d*) A single release at a 1 : 2 ratio of a strain that is homozygous for two insertions of K and one insertion of R. Dash-dotted line, wild-type (kkrr); dotted line, K allele; dashed line, R allele; solid line, mean fitness.

**Figure 4:**
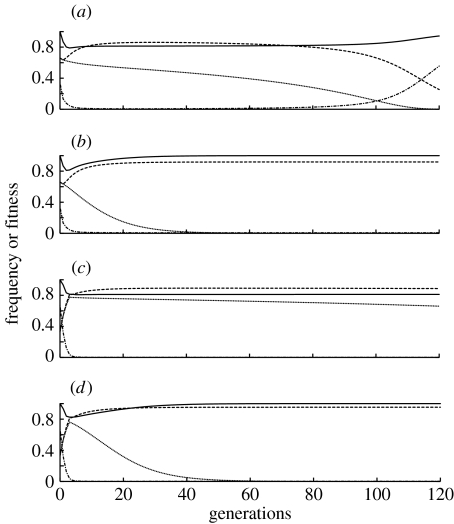
Allele frequencies, wild genotype frequencies and population fitness over time, following the release of killer–rescue strains with either *c*_K2_ or *c*_R2_ being equal to 0.20 and the other being 0. (*a*) Single 2 : 1 release with fitness cost *c*_R2_=0.20, and *c*_K2_=0. (*b*) Single 2 : 1 release with fitness cost *c*_K2_=0.20, and *c*_R2_=0. (*c*) Four sequential releases, each at a 1 : 2 ratio with fitness cost *c*_R2_=0.20, and *c*_K2_=0. (*d*) Four sequential releases, each at a 1 : 2 ratio with fitness cost *c*_K2_=0.20, and *c*_R2_=0. In each case, the fitness costs are additive, with one insertion of K and R, and a 1 : 1 release sex ratio. Dash-dotted line, wild-type (kkrr); dotted line, K allele; dashed line, R allele; solid line, mean fitness.

**Figure 5:**
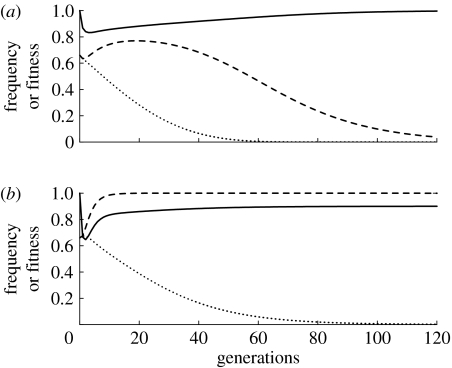
Allele frequencies and population fitness over time, following the release of killer–rescue strains with incomplete killing or non-dominant inheritance of rescue. (*a*) Effect of the killer gene causing 50% mortality of all individuals lacking an R allele (results are identical if all females without R die, but no males die); complete dominance of killing and rescue. (*b*) Dynamics when KkrR and KKRR individuals have 0% mortality but individuals with KkRr or KKRr have 50% mortality (additive inheritance of rescue). Dotted line, K allele; dashed line, R allele; solid line, mean fitness.

## Supplementary Material

Mathematical analysis and additional scenarios
