# Supplementary material for: A Killer–Rescue system for self-limiting gene drive of anti-pathogen constructs
Source: Proc Biol Sci. 2008 Sep 2;275(1653):2823–9. doi: 10.1098/rspb.2008.0846 (PMC2572679; doi:10.1098/rspb.2008.0846)
Supplement: Supplementary file 1 [file rspb20080846s06.doc]

# SUPPLEMENTAL MATERIALS

I. Time Series Dynamics with Dominant and Recessive Fitness Costs
II. Time Series Dynamics of All Male Release
III. Allele Fitnesses Over Time
IV. Iterative equations for the two-locus killer-rescue system
V. Equilibrium and local stability analysis
   A. No fitness costs
   B. Cost for Rescue alone (*c*_K1_ = *c*_K2_ = 0, *c*_R2_ > 0)
   C. Cost for Killer allele alone (*c*_R1_ = *c*_R2_ = 0, *c*_K2_ > 0)
   D. Costs to both Killer and Rescue.
VI. Allele Frequency Trajectories
VII. Introduction threshold analysis for the no fitness costs case and the dominant rescue-only cost case

# I. Time Series Dynamics with Dominant and Recessive Fitness Costs

Although in the absence of empirical data it is reasonable to assume additive fitness costs, there are a number of sources of fitness costs that could result in more dominant or more recessive fitness costs. For example, a fitness cost due to the site of insertion could be recessive if only homozygosity of the specific gene disrupted by the insertion of the construct, or homozygosity of the region flanking the insertion, leads to unfit phenotypes. More dominant acting fitness effects could be seen if expression of one copy of the anti-pathogen gene decreased fitness about the same amount as expression of two copies.

We compared the results shown in Figure 3a (fitness costs are additive, *c*_K2_ and *c*_R2_ both equal 0.10, and a single 2:1 release) with results from similar releases except that the fitness cost associated with either K or R was changed to be completely recessive or completely dominant. (We retained an additive interaction between the two loci, so there is no epistasis.)

Changing the dominance of the fitness cost associated with K (Figure S1a and S1b) while holding the R fitness cost as additive had only a small impact on the results. However, when the K fitness cost was held as additive and the dominance of the R fitness cost was varied (Figure S1c and S1d) a stronger effect was seen. When *c*_R1_ equals *c*_R2_ (complete dominance of R), the frequency of R remains very high through generation 120. This very slow decline in R is attributable to the fact that there is very low additive genetic variance for fitness when the frequency of R is high and individuals with RR and Rr have the same fitness. The most positive result in terms of sustaining low transmission of the pathogen occurs if the K fitness cost is completely recessive and the R fitness cost is completely dominant. In this case the R allele remains at high frequency past generation 600 (not shown).

**Figure S1. Allele frequencies and population fitness over time, following the release of Killer-Rescue strains with *c*_K2_ and *c*_R2_ each equal to 0.1 and with varying dominance of the fitness costs.** All releases are at a 2:1 ratio. a) The fitness cost associated with K is recessive and the fitness cost associated with R is additive. b) The K fitness cost is dominant and the R fitness cost is additive. c) The K fitness cost is additive and the R fitness cost is recessive. d) The K fitness cost is additive and the R fitness cost is dominant.

**II. Time Series Dynamics of All Male Release**

In all of the model runs above and in the main text, the releases involved a 1:1 sex ratio of engineered mosquitoes. Even though the mosquitoes to be released are homozygous for a gene that interrupts transmission of the pathogen, regulations may only allow release of male mosquitoes because excess males do not increase population growth, and they do not bite humans or animals. We therefore ran the model with releases of only males. The ratios of released males to the entire native population of males and females were either 2:1 or 1:2. We assumed both *c*_K2_ and *c*_R2_ were equal to 0.10 and additive inheritance, as in the results presented in Figure 3. A single 2:1 release of males (Figure S2a) is not as effective as a 2:1 release that includes both males and females (Figure 3a). However, four releases of all males in a 1:2 ratio to the natural population (Figure S2b) gave results similar to four releases of males and females in a 1:2 ratio (Figure 3c).

When only one sex is released, all of the released individuals must mate with wildtype individuals of the opposite sex. No matter how high the release level, even at a ratio of 19:1, the K and R frequency in the first generation can only approach 0.5. As the ratio of released males to native males and females increases, the efficiency of the release goes down. If a given number of males is released over a few generations instead of one generation, the efficiency is decreased to a lesser extent.

Figure S2. Allele frequencies and population fitness over time, following release of Killer-Rescue strain males. Additive fitness costs with *c*_K2_ and *c*_R2_ each equal to 0.1. a) Single 2:1 release of males relative to all males and females in the wild population. b) Four 1:2 releases of all males.

### III. Allele Fitnesses

In two-locus systems with inter-locus interactions (epistasis), the selection factors responsible for changes in allele frequencies over time are not always intuitive. We, therefore, found it useful to plot the overall fitness of each allele (K, k, R and r) over time as the genotype frequencies in the population changed. The relative fitness of each allele per generation is defined as the ratio of the number of copies of the allele expected when the Killing gene is active to the expected number of copies of that allele if the Killing gene was made inactive (fitness costs remaining). At a qualitative level it is clear that the fitness of the r allele in a population is lower when the frequency of the K allele is 0.9 than when the K allele has a frequency of 0.1. For a given frequency of K, the r allele fitness is higher when the frequency of R is high and the r allele is therefore often imbedded in a rescued Rr heterozygote genotype.

We have plotted the allele fitnesses to parallel the allele frequency changes in the scenarios of Figures 3a,b,c, and d, where *c*_K2_ and *c*_R2_ are both 0.10, and have additive effects. In this case, when the initial release frequency is 0.33 (Figure 3b and Figure S3b) the Rescue allele (R) and the non-rescue allele (r) have equal fitness at generation 12 (Figure S3b). At this point Figure 3b shows that the frequency of allele R stops increasing. This same dynamic is found in the case with insertion of the K allele at two loci (Figure 3d and Figure S3d) except that it takes longer for the fitness of r to reach that of R (~40 generations).

The lowest fitness of the K allele is seen with an initial release of 0.33 (Figure S3b) when the frequency of K is very low and R is decreasing. This is reasonable because as the frequency of R approaches zero, the fitness of the K allele approaches 0.0.

**Figure S3.** Fitnesses of alleles K, k, R and r alleles over time when release frequencies and engineered strain properties are as in figure 3 of the main text. In all cases *c*_K2_ and *c*_R2_ are both 0.10, and have additive effects.

**IV. Iterative equations for the two-locus killer-rescue system**

Our mathematical model for the two-locus killer-rescue system is most succinctly represented as a set of iterative equations for the gamete frequencies. We write the frequencies of the four gametes as follows: *x*_1_ stands for the frequency of KR, *x*_2_ denotes the frequency of Kr, *x*_3_ denotes the kR frequency and *x*_4_ denotes the frequency of kr. For each of these we have 0 ≤ *x_i_* ≤ 1. We write the fitness cost to a genotype heterozygous for R as *c*_R1_, the cost to a homozygote for R as *c*_R2_, the cost to a genotype heterozygous for K as *c*_K1_, and the cost to a homozygote for K as *c*_K2_. We have that 0 ≤ *c*_K1_ ≤ *c*_K2_ and 0 ≤ *c*_R1_ ≤ *c*_R2_.

We assume that fitness costs are additive between the two loci. (a separate analysis that assumed multiplicative interactions produced similar results.) This imposes the constraint that
1 – *c*_R_*_i_* - *c*_K_*_j_* ≥ 0 for all pairs ( *i* , *j* ), since fitnesses cannot be negative. Here we assume that both killer and rescue are completely dominant: genotypes carrying no rescue allele but at least one killer allele are not viable, whereas genotypes carrying at least one rescue allele are viable.

We obtain the following equations for the gamete frequencies in the next generation (denoted by primes) in terms of the current generation’s gamete frequencies

$\begin{aligned} \text{Wx}_{1}^{'}=(1-c_{R2}-c_{K2})x_{1}^{2}+(\text{1-}c_{\text{R1}}-c_{K2})x_{1}x_{2}+(1-c_{R2}-c_{\text{K1}})x_{1}x_{3}+\frac{1}{2}(\text{1-}c_{\text{R1}}-c_{\text{K1}})x_{1}x_{4}+\frac{1}{2}(\text{1-}c_{\text{R1}}-c_{\text{K1}})x_{2}x_{3} \\ \text{Wx}_{2}^{'}=(\text{1-}c_{\text{R1}}-c_{K2})x_{1}x_{2}+\frac{1}{2}(\text{1-}c_{\text{R1}}-c_{\text{K1}})x_{1}x_{4}+\frac{1}{2}(\text{1-}c_{\text{R1}}-c_{\text{K1}})x_{2}x_{3} \\ \text{Wx}_{3}^{'}=(1-c_{R2}-c_{\text{K1}})x_{1}x_{3}+\frac{1}{2}(\text{1-}c_{\text{R1}}-c_{\text{K1}})x_{1}x_{4}+\frac{1}{2}(\text{1-}c_{\text{R1}}-c_{\text{K1}})x_{2}x_{3}+(1-c_{R2})x_{3}^{2}+(\text{1-}c_{\text{R1}})x_{3}x_{4} \\ \text{Wx}_{4}^{'}=\frac{1}{2}(\text{1-}c_{\text{R1}}-c_{\text{K1}})x_{1}x_{4}+\frac{1}{2}(\text{1-}c_{\text{R1}}-c_{\text{K1}})x_{2}x_{3}+(\text{1-}c_{\text{R1}})x_{3}x_{4}+x_{4}^{2}\text{.} \end{aligned}$	(1)

Here, *W* is the mean fitness of the gametes, whose value is given by the sum of all of the terms on the right-hand sides of the four equations. (If there were no fitness costs and all genotypes were viable then *W* would equal one, but in general its value equals one minus the sum of all the reductions in contributions to the next generation due to fitness costs and non-viability of genotypes. In this light, we see that the expression for *W* is more complicated when there are fitness costs than when they are absent. As we shall see below, this impacts our ability to obtain analytic expressions for equilibria.) Since we have that *x*_1_ + *x*_2_ + *x*_3_ + *x*_4_ = 1, one of the above equations is redundant: we can eliminate any one of the *x_i_*, rewriting our system as a three dimensional model.

The corresponding equations under the assumption of multiplicative fitness costs between the two loci can be obtained by a straightforward modification of the above set of equations.

# V. Equilibrium and local stability analysis

The behavior of the model is investigated using a combination of equilibrium and local stability analysis, aided by the computer algebra package Maple (Maplesoft, Waterloo Ontario, Canada) and numerical simulation. We cannot find complete analytic solutions for all equilibria for general values of the parameters describing the fitness costs so we instead concentrate on special cases that cover many of the situations of greatest interest.

It is straightforward to show that the three lines *x*_1_ + *x*_3_ = 1 (i.e. a mixture of KR and kR alone: the rescue allele, R, is fixed), *x*_3_ + *x*_4_ = 1 (i.e. a mixture of kR and kr alone: the killer allele, K, is absent), and *x*_1_ + *x*_2_ = 1 (i.e. a mixture of KR and Kr: the killer allele, K, is fixed) are invariant. Trajectories that start on any one of these lines remain there. Points on the line *x*_2_ + *x*_4_ = 1 get mapped onto the point (0, 0, 0, 1): if there is a mixture of Kr and kr alone, then the following generation contains just kr (both K and R absent).

The existence of these invariant lines means that the dynamics of the system on each of these lines can be described by a single equation.

On the “R fixed” line, *x*_1_ + *x*_3_ = 1, the governing equations can be shown to reduce to the following equation

$x_{3}^{'}=\left( \frac{1-c_{R2}-c_{K1}+c_{\text{K1}}x_{3}}{1-c_{\text{R2}}-c_{\text{K2}}+2\left( c_{\text{K2}}-c_{\text{K1}} \right)x_{3}+\left( 2c_{\text{K1}}-c_{\text{K2}} \right)x_{3}^{2}} \right)x_{3}\text{.}$	(2)

(Alternatively, an equation can be derived to describe how the *x*_1_ component changes.)

On the “K absent” line, *x*_3_ + *x*_4_ = 1, the system can be reduced to

$x_{3}^{'}=\left( \frac{1-c_{\text{R1}}+\left( c_{R1}-c_{\text{R2}} \right)x_{3}}{1-2c_{\text{R1}}x_{3}+\left( 2c_{R1}-c_{\text{R2}} \right)x_{3}^{2}} \right)x_{3}\text{.}$	(3)

Finally, on the “K fixed” line, *x*_1_ + *x*_2_ = 1, the system reduces to

$x_{1}^{'}=\frac{1-c_{\text{R1}}-c_{\text{R2}}+\left( c_{\text{R1}}-c_{\text{R2}} \right)x_{1}}{2\left( 1-c_{\text{R1}}-c_{K2} \right)+\left( 2c_{\text{R1}}-c_{R2}+c_{K2}-1 \right)x_{1}}\text{.}$	(4)

# A. No fitness costs

There are two lines of equilibria:

1. **R fixed**: *x*_1_ + *x*_3_ = 1,
2. **K absent**: *x*_3_ + *x*_4_ = 1,

and two isolated equilibria:

3. (*b*, *b*, *-b*, 1-*b*), where $b=1\pm\frac{1}{2}\sqrt{2}$. These equilibria are not biologically feasible since the second and third components have opposite signs: one component must be negative.

Local linear stability analysis proceeds in the usual fashion: the 3×3 Jacobian matrix of the equations relating the gamete frequencies in successive generations is calculated and evaluated at the equilibrium point of interest. The eigenvalues of this matrix are calculated and their moduli examined. If all eigenvalues have modulus less than one then the equilibrium is (locally) stable. If one or more eigenvalue has modulus greater than one then the equilibrium is unstable. Linear stability analysis is inconclusive if the eigenvalue with largest modulus falls on the unit circle: a nonlinear analysis, which may be quite involved, is required to determine stability in such instances.

The Jacobian matrix of the system evaluated at any point on the “R fixed” line has eigenvalues equal to 1, 1 and ½. One eigenvalue of unity arises because there is no motion along the line of equilibria: there is neutral stability along the line. Numerical simulation indicates that the line is attracting, but, as the second eigenvalue equals one, linear stability analysis is inconclusive. The nonlinear analysis required to prove that this line is attracting is not straightforward. (Stability properties could be explored by way of a center manifold reduction, but we have not pursued this.)

The Jacobian matrix evaluated at points on the “K absent” line has eigenvalues 1 and $\frac{1}{4}\left( 1+2x_{3}\pm\sqrt{1+4x_{3}-4x_{3}^{2}} \right)$. Again, the first eigenvalue corresponds to neutral stability along the direction of the line. The remaining two eigenvalues are easily seen to have modulus less than or equal to one for 0 ≤ *x*_3_ ≤ 1, with the larger of the two attaining the value of one when *x*_3_ = 1. For points with 0 ≤ *x*_3_ < 1, the line is attracting. We note that *x*_3_ = 1 corresponds to the point of intersection of the “R fixed” and “K absent” lines of equilibria, which occurs at the point (0, 0, 1, 0), where R is fixed and K is absent.

Since the wild-type population has genotype kkrr and our introduced individuals have genotype KKRR, the initial conditions to be considered correspond to a pure mixture of kr and KR gametes, i.e. the line *x*_1_ + *x*_4_ = 1. (In all of what follows, we use the term “initial conditions” to denote points on this line, rather than a general initial condition of the system.) In the long run, numerical simulation shows that trajectories started from initial conditions on this line approach equilibria that are either on the “R fixed” or “K absent” lines. One such trajectory approaches the intersection point of these lines (i.e. the point where R is fixed and K is absent): the initial condition that gives rise to this trajectory represents a threshold introduction level that separates these two outcomes. An analytic calculation of this threshold appears to be difficult, given that it requires the calculation of a complete trajectory of the nonlinear system. Numerical simulation gives this threshold introduction level as approximately *x*_1_=0.350.

Figure S4. summarizes the behavior of the system in this case. The initial conditions lie on the solid black line. The gamete frequencies in successive generations are depicted as dots, with lines joining the dots to aid visualization of the trajectories. The “R fixed” line of equilibria is shown as a solid green line (points on this line represent mixtures of KR and kR), and the “K absent” line of equilibria as a solid red line (points on this line represent mixtures of kR and kr). Trajectories are colored red or green, according to which of these two outcomes occurs in the long term.

Figure S4. Trajectories of the system in the no cost case. As discussed in the text, the gamete frequencies in successive generations are depicted as dots, with lines joining the dots to aid visualization of the trajectories. The “R fixed” line of equilibria is shown as a solid green line (points on this line represent mixtures of KR and kR), and the “K absent” line of equilibria as a solid red line (points on this line represent mixtures of kR and kr). Trajectories are colored red or green, according to which of these two outcomes occurs in the long term. Initial conditions lie on the solid black line.

**B. Cost for Rescue alone (*c*_K1_ = *c*_K2_ = 0 , *c*_R2_ > 0)**

In this case, analytic expressions for the equilibria can be found for all values of *c*_R1_ and *c*_R2_.

1. **R fixed** line of equilibria: *x*_1_ + *x*_3_ = 1,
2. **Both K and R absent** equilibrium point: (0, 0, 0, 1), i.e. kr only. (This is an endpoint of the K absent line from the previous case.)
3. **KR/Kr mixture** equilibrium point:$\left( \frac{1-c_{\text{R1}}}{1-2c_{\text{R1}}+c_{\text{R2}}},\frac{c_{\text{R2}}-c_{\text{R1}}}{1-2c_{\text{R1}}+c_{\text{R2}}},0,0 \right)$. This isolated equilibrium has KR and Kr alone: the K allele is fixed. If *c*_R1_ = *c*_R2_ then this equilibrium has just KR (it is an endpoint of the R fixed line).
4. (*b*, *b*, -*b*, 1*-b*), where *b* = $1\pm\frac{1}{2}\sqrt{2-2c_{\text{R1}}}$. It is easy to see that these two equilibria are not biologically feasible, since *b* is non-zero when 0 ≤ *c*_R1_ ≤ 1.

The eigenvalues for points on the “R fixed” line of equilibria are 1, (1- *c*_R1_)/(1- *c*_R2_) and
(1/2)(1- *c*_R1_)/(1- *c*_R2_). The first eigenvalue corresponds to neutral stability along the direction of the line. There are two possible cases: (a) *c*_R2_ > *c*_R1_, or *c*_R2_ = *c*_R1_.

(a) If *c*_R2_ > *c*_R1_, then the second eigenvalue is greater than one and the equilibria are unstable.

(b) If *c*_R2_ = *c*_R1_, then the second eigenvalue equals one and the third eigenvalue equals ½: linear stability analysis is inconclusive in this case, although numerical simulation suggests that the line is attracting. As before, it may be possible to carry out a nonlinear analysis to verify this, likely by way of a center manifold approach, but we do not pursue this here.

The eigenvalues for the “both K and R absent” equilibrium are 0, 1-*c*_R1_ and (1- *c*_R1_)/2. There are two possible cases: (a) *c*_R1_ > 0, or (b) *c*_R1_ = 0.

(a) This equilibrium is stable when *c*_R1_ > 0.

(b) Linear stability analysis is inconclusive when *c*_R1_ = 0, but the following nonlinear analysis shows that the equilibrium remains stable. We consider a small region inside the biologically relevant region that is close to the equilibrium point, so that *x*_1_, *x*_2_ and *x*_3_ are all small. We can show that trajectories approach the “K absent” line, *x*_3_ + *x*_4_ = 1, by considering the distance of successive iterates from this line: this distance is given by *x*_1_^2^ + *x*_2_^2^. The distance of the next iterate from the line, i.e. *x*_1_′ ^2^ + *x*_2_′ ^2^, can then be calculated using the governing equations. A second order Taylor series expansion in *x*_1_, *x*_2_ and *x*_3_ gives this new distance (to leading order) as ½*x*_1_^2^: successive iterates indeed move closer to the line if *x*_1_ > 0. If *x*_1_ = 0, we have to retain more terms in our Taylor expansion; the first non-zero term is ½*x*_2_^2^*x*_3_^2^ and so we see iterates move closer to the line in this case also. Points on the “K absent” line, *x*_3_ + *x*_4_ = 1, can be seen to approach the “both K and R absent” equilibrium, (0, 0, 0, 1). For a point on the “K absent” line, the dynamics are described by equation (3), which, in this case, gives

$$x_{3}^{'}=\frac{1-c_{\text{R2}}x_{3}}{1-c_{\text{R2}}x_{3}^{2}}x_{3}\text{.}$$

For *c*_R2_ > 0 and 0 < *x*_3_ < 1, the term multiplying *x*_3_ is less than one, so *x*_3_ decreases towards zero: points move towards the equilibrium. The equilibrium, therefore, is seen to be stable.

The “KR/Kr mixture” equilibrium has eigenvalues 1, (1- *c*_R2_)/ (1- *c*_R1_) and (1/2)(1- *c*_R2_)/ (1- *c*_R1_). Recall that this equilibrium is of interest when *c*_R2_ > *c*_R1_ (otherwise it is an endpoint of the “R fixed” line of equilibria already discussed). We notice that the second eigenvalue is the reciprocal of the second eigenvalue of equilibrium 1: it is less than one if *c*_R2_ > *c*_R1_ and equals one when *c*_R2_ = *c*_R1_. The third eigenvalue is always less than one. Linear stability analysis is inconclusive, but numerical simulations suggest that the equilibrium is unstable in the *c*_R2_ > *c*_R1_ case of interest.

The phase portrait looks quite different depending on whether we have *c*_R2_ = *c*_R1_ (figure S5, dominant fitness cost) or *c*_R2_ > *c*_R1_ (figure S6). When the fitness cost is dominant, there are two possible outcomes, with either R becoming fixed or R being lost. There is a threshold introduction level of KR that separates these two outcomes, with the level of the threshold depending on the fitness cost. As the fitness cost approaches zero, the threshold value approaches that seen in the no cost case. When the fitness cost is not dominant (figure 3), we see that almost every trajectory from our initial conditions leads to the equilibrium at which both K and R are lost (i.e. kr only).

**Figure S5. Trajectories of the system when there are strictly dominant costs to the Rescue allele, but no costs to the Killer allele.** Rescue costs are given by *c*_R2_ = *c*_R1_ = 0.1. There is a stable equilibrium at which both Killer and Rescue are absent, i.e. all kr, shown by the filled magenta circle. The “Rescue fixed” line of equilibria (i.e. points are mixtures between kR and KR) is shown in green. A trajectory starting at any the initial conditions either approaches the “both K and R absent” (all kr) equilibrium or the “Rescue fixed” line of equilibria. Trajectories are colored to indicate their eventual fate. The position of the threshold that separates these two types of trajectories depends on the value of the cost. For higher costs, the threshold moves further away from the “both K and R absent” (all kr) equilibrium. As the cost approaches zero, the threshold moves towards the value seen in the earlier no cost case.

**Figure S6. Trajectories of the system when there are costs to the Rescue allele, with *c*_R2_ > *c*_R1_, but no costs to the Killer allele.** Rescue costs are given by *c*_R1_ = 0, *c*_R2_ = 0.1. There is a stable equilibrium at which both Killer and Rescue are absent, i.e. all kr, shown by the filled magenta circle. There is an unstable fixed point (the “KR/Kr mixture” equilibrium) on the KR axis, shown as an unfilled black circle. The “Rescue fixed” line of equilibria (i.e. points are mixtures between kR and KR) is shown in green; this line is repelling. Except for the initial condition with all KR, trajectories starting at all other initial conditions approach the all kr equilibrium. These trajectories are seen to approach a curve as they move towards the all kr equilibrium. This curve appears to originate at the unstable equilibrium on the KR axis.

**C. Cost for Killer allele alone (*c*_R1_ = *c*_R2_ = 0, *c*_K2_ > 0)**

For general choices of *c*_K1_ and *c*_K2_, the following equilibria were found:

1. **Both K and R fixed** equilibrium point: (1, 0, 0, 0), i.e. all KR,
2. **K absent** line of equilibria: *x*_3_ + *x*_4_ = 1,
3. **KR/kR mixture** equilibrium point: $\left( \frac{c_{\text{K1}}}{2c_{\text{K1}}-c_{\text{K2}}},0,\frac{c_{K1}-c_{\text{K2}}}{2c_{\text{K1}}-c_{K2}},0 \right)$
   There are three possible cases: (a) 0 < *c*_K1_ < *c*_K2_, (b) 0 < *c*_K1_ = *c*_K2_, or (c) 0 = *c*_K1_ < *c*_K2_.
   (a) If *c*_K1_ is positive and not equal to *c*_K2_, this equilibrium is not biologically relevant since the product of the first two components is equal to $c_{\text{K1}}(c_{K1}-c_{\text{K2}})/\left( 2c_{\text{K1}}-c_{\text{K2}} \right)^{2}$. The value of this product is negative, since *c*_K2_ > *c*_K1_, and so one component of the equilibrium must be negative.
   (b) Two cases remain: if *c*_K1_ is positive and equals *c*_K2_, we see that this equilibrium coincides with the “both K and R fixed” equilibrium.
   (c) If *c*_K1_ equals zero, this equilibrium is at (0, 0, 1, 0), i.e. K absent and R fixed. This point coincides with an endpoint of the “K absent” line of equilibria.
   Taken together, (a), (b) and (c) mean that we need not consider this equilibrium separately.
4. Four further equilibria exist. A quartic polynomial can be found whose four solutions are the values taken by one component (e.g. *x*_2_) of the equilibrium. The values of the remaining components can be given in terms of this component, although these expressions are complicated. In general, this polynomial does not have a simple solution and, given the complexity of the expression for the equilibria (the output from Maple contains almost 100 000 terms) we do not show the solution here. Numerical exploration of these solutions suggests that they are often biologically irrelevant since once or more components are often either negative or complex.

The eigenvalues of the Jacobian matrix evaluated at the “both K and R fixed” equilibrium are 1, $\frac{1-c_{\text{K1}}}{1-c_{\text{K2}}}$ and $\frac{1}{2}\left( \frac{1-c_{\text{K1}}}{1-c_{\text{K2}}} \right)$. There are two possible cases: (a) *c*_K2_ > *c*_K1_, or (b) *c*_K2_ = *c*_K1_.

(a) The second eigenvalue is greater than one if *c*_K2_ > *c*_K1_: the equilibrium is unstable.

(b) If *c*_K2_ = *c*_K1_, linear stability analysis is inconclusive, but a simple analysis shows that the equilibrium is again unstable. Writing the common cost as *c*_K_, use of equation (2) shows that the dynamics on the invariant “R fixed” line are given by

$$x_{3}^{'}=\left( \frac{1-c_{K}+c_{K}x_{3}}{1-c_{K}+c_{K}x_{3}^{2}} \right)x_{3}\text{.}$$

The factor in parentheses that multiplies *x*_3_ is greater than one for 0 < *x*_3_ < 1, so the value of *x*_3_ increases. In particular, successive iterates move away from the “both K and R fixed” equilibrium at (1, 0, 0, 0). The equilibrium point is, therefore, unstable. (More specifically, we see that the value of *x*_3_ will approach one: points on the “R fixed” line approach the “K absent and R fixed” point, i.e. the intersection of the “R fixed” invariant line and the “K absent” line of equilibria.)

The eigenvalues of the Jacobian matrix evaluated at points on the “K absent” line are 1 and $\frac{1-c_{\text{K1}}}{4}\left( 1+2x_{3}\pm\sqrt{1+4x_{3}-4x_{3}^{2}} \right)$. There is neutral stability along the direction of the line and the remaining two eigenvalues have modulus less than or equal to one for 0 ≤ *x*_3_ ≤ 1, with the larger attaining the value of one if *x*_3_ equals one and *c*_K1_ equals 0. For points with 0 ≤ *x*_3_ < 1, the line is attracting.

The qualitative behavior of the system is the same in all cases here: provided that the initial condition is not at the unstable “all KR” equilibrium, the trajectory approaches the “K absent” line, *x*_3_ + *x*_4_ = 1: the killer allele is lost (figure S7).

**Figure S7. Trajectories of the system when there are costs to the Killer allele but not to the Rescue allele.** *c*_K1_ = 0, *c*_K2_ = 0.05, *c*_R1_ = *c*_R2_ = 0. There is an unstable “all KR” (K and R both fixed) equilibrium (open green circle) and an attracting “K absent” line of equilibria (solid red line, points on which are a mixture of kR and kr). All trajectories that start away from the unstable equilibrium approach the “K absent” line.

D. Costs to both Killer and Rescue. Costs taken to be additive between loci.

The difficulties we had in finding analytic solutions in the Killer cost only case were compounded here. Maple was unable to find all equilibrium solutions for general values of the fitness costs. We did find, however, three equilibria that were common to all cases:

1. **Both K and R absent** equilibrium: (0, 0, 0, 1), i.e. all kr.
2. **K absent and R fixed** equilibrium: (0, 0, 1, 0), i.e. all kR.
3. **Both K and R fixed** equilibrium: (1, 0, 0, 0), i.e. all KR.

We shall first discuss the stability of these equilibria.

# The equilibrium at which both K and R are absent has eigenvalues 0, 1-*c*_R1_ and (1- *c*_R1_- *c*_K1_)/2.

# (a) If *c*_R1_ > 0, this equilibrium is stable.

# (b) When *c*_R1_ = 0, the second eigenvalue equals one and so the linear stability analysis is inconclusive. The eigenvector corresponding to this eigenvalue is in the direction of the “K absent” invariant line, *x*_3_ + *x*_4_ = 1, so to show stability in this case we must demonstrate that points on this line move towards the equilibrium. As before, equation (3) shows that the governing equations reduce to a single equation for one component

$$x_{3}^{'}=\left( \frac{1-c_{R2}x_{3}}{1-c_{R2}x_{3}^{2}} \right)x_{3}\text{.}$$

For 0 < *x*_3_ < 1, we see that the factor in parentheses multiplying *x*_3_ is less than one, i.e. the *x*_3_ component of successive iterates of points on the line decreases. Iterates thus move towards the “both K and R absent” equilibrium, (0, 0, 0, 1), which is therefore stable.

# The equilibrium at which K is absent and R is fixed has eigenvalues $\frac{1-c_{R2}-c_{\text{K1}}}{1-c_{\text{R2}}}$, $\frac{1-c_{\text{R1}}}{1-c_{\text{R2}}}$and $\frac{1}{2}\left( \frac{1-c_{\text{R1}}-c_{K1}}{1-c_{R2}} \right)$. There are two possible cases: either (a) *c*_R2_ > *c*_R1_, or (b) *c*_R2_ = *c*_R1_.

# (a) The second eigenvalue is greater than one whenever *c*_R2_ > *c*_R1_, in which case the equilibrium is unstable.

# (b) If *c*_R2_ = *c*_R1_ we can see that the equilibrium remains unstable using the earlier analysis of behavior on the “K absent” invariant line, *x*_3_ + *x*_4_ = 1. Writing the common value of *c*_R1_ and *c*_R2_ as *c*_R_, we get

$$x_{3}^{'}=\left( \frac{1-c_{R}}{1-2c_{R}x_{3}+c_{R}x_{3}^{2}} \right)x_{3}\text{.}$$

The factor in parentheses is less than one for 0 < *x*_3_ < 1, so the *x*_3_ component decreases: points on the line move away from the “K absent and R fixed” equilibrium, (0, 0, 1, 0), which is therefore unstable.

# The equilibrium for which both K and R are fixed has eigenvalues $\frac{1-c_{R2}-c_{K1}}{1-c_{R2}-c_{K2}}$, $\frac{1-c_{R1}-c_{K2}}{1-c_{R2}-c_{K2}}$ and $\frac{1}{2}\left( \frac{1-c_{\text{R1}}-c_{\text{K1}}}{1-c_{\text{R2}}-c_{\text{K2}}} \right)$. The first eigenvalue is greater than one if *c*_K2_ > *c*_K1_, and equals one if these two costs are equal. Similarly, the second eigenvalue is greater than one if *c*_R2_ > *c_­_*_R1_, and equals one if these two costs are equal. This means that this equilibrium is guaranteed to be unstable unless both fitness costs are dominant. If both fitness costs are dominant, the equilibrium can still be seen to be unstable by considering the behavior on the “R fixed” invariant line, *x*_1_ + *x*_3_ = 1. Equation (2) gives

$$x_{3}^{'}=\left( \frac{1-c_{R}-c_{K}+c_{K}x_{3}}{1-c_{R}-c_{K}+c_{K}x_{3}^{2}} \right)x_{3}\text{.}$$

Here *c*_R_ is the cost for being either heterozygous or homozygous for R and *c*_K_ is the corresponding cost for K; both of these constants are positive. For 0 < *x*_3_ < 1, we see that the factor in parentheses multiplying *x*_3_ is greater than one, which means that points on the line move away from the “both K and R fixed” equilibrium, (0, 0, 0, 1), which is therefore unstable.

There are other equilibria beyond these three. We only managed to make progress on locating their position in the special case in which *c*_K1_ = *c*_K2_ = *c*_R1_ = *c*_R2_, i.e. equal, dominant fitness costs for Killer and Rescue, when we obtain

4. Six further equilibria, found in terms of the solution of a sixth degree polynomial. (The polynomial is not as complex as in the general killer cost case, but perhaps this is unsurprising as we only have one independent parameter here but had two there.)

When there are fitness costs for both killer and rescue, taken to be additive between loci, trajectories started at almost every initial condition (i.e. except for the unstable “both K and R fixed” equilibrium) approach the “K and R both absent” equilibrium, i.e. both killer and rescue are lost (figure S8). Ultimately, trajectories approach this equilibrium along the “K absent” line (i.e. the killer allele has been lost first), although some trajectories—those with a sufficiently high initial frequency of KR—first approach and then move along the “R fixed” line before doing so (i.e. R comes close to fixation, but does not attain it, only to be lost later on).

**Figure S8. Behavior of the system when both killer and rescue alleles have a cost.** Parameter values are *c*_R1_ = 0.2, *c*_R2_ = 0.2, *c*_K1_ = 0.005, and *c*_K2_ = 0.005. The “both K and R fixed” equilibrium (open black circle) is unstable, as is the “R fixed and K absent” equilibrium (open green circle). The “both K and R absent” equilibrium (all kr: filled magenta circle) is stable. Trajectories from all initial conditions (except the “all KR” equilibrium) approach the “both K and R absent” (i.e. all kr) equilibrium: both killer and rescue alleles are lost.

**VI. Allele Frequency Trajectories**

A convenient alternative formulation of the model, mathematically equivalent to the iterative equations given above, is framed in terms of the frequencies of the K and R alleles, which we write as *q* and *p*, and the linkage disequilibrium, *D*. These three quantities can be written in terms of the gamete frequencies as follows (see, for example, Hartl and Clark 1997)
$\begin{aligned} p=x_{1}+x_{3} \\ q=x_{1}+x_{2} \\ D=x_{1}x_{4}-x_{2}x_{3}\text{.} \end{aligned}$

In turn, the allele frequencies can be expressed in terms of *p*, *q* and *D*:

$$\begin{aligned} x_{1}=\text{pq}+D \\ x_{2}=\left( 1-p \right)q-D \\ x_{3}=p\left( 1-q \right)-D \\ x_{4}=\left( 1-p \right)\left( 1-q \right)+D\text{.} \end{aligned}$$

The iterative equations for the allele frequencies can be rewritten to give iterative equations for *p*, *q* and *D*. If there are no fitness costs, the following set of equations is obtained

$\begin{aligned} p^{'}=\frac{p}{W} \\ q^{'}=\frac{\text{pq}\left( 2-p \right)+D\left( 1-p \right)}{W} \\ D^{'}=\frac{W\left( 2\text{pq}+D \right)+2\text{pD}\left( p-1 \right)+2p^{2}q\left( p-2q \right)}{2W^{2}}\text{.} \end{aligned}$	(5)

Here, *W* is the mean fitness, which equals 1-2*x*_2_*x*_4_-*x*_2_^2^ in terms of gamete frequencies, or
1-[(1-*p*)*q*-*D*][(1-*p*)(2-*q*)+*D*] in terms of allele frequencies.

Earlier we depicted trajectories of the system using three-dimensional gamete frequency plots. Equivalent plots in (*p*, *q*, *D*) space could be presented, but almost all the information presented by such figures can be conveyed using simpler two-dimensional plots that depict the allele frequency trajectories in the (*q*, *p*) plane.

The allele frequency trajectories in the no cost case are presented in Figure S9. Because KKRR homozygotes are introduced, the initial point of each trajectory has *p* equal to *q*, and so lies on the diagonal line *p* = *q*, which we depict as a solid black line. Initial conditions and trajectories that ultimately lead to fixation of R are shown in green, and those that lead to the extinction of K are shown in red.

**Figure S9. (Shown as Figure 2 in the main text.) Allele frequency trajectories following release of Killer-Rescue homozygotes at a number of different initial frequencies.** The fitness costs of K and R are set at zero. The allele frequencies in successive generations are depicted as dots, with lines joining the dots to aid visualization of the trajectories. (The closer dots are along a line, the less change there is in allele frequencies per generation, so, as equilibrium is approached, the dots appear to overlap). The R fixed line of equilibria is shown as a solid green line, and the K absent line of equilibria as a solid red line. Trajectories are colored red or green, according to which of these two outcomes occurs in the long term. Initial conditions lie on the solid black line.

For completeness, we present plots (Figures S10 through S13) of allele frequency trajectories corresponding to the fitness costs depicted in Figures S5 through S8. In each case we see that both allele frequencies change by the same amount in the first generation: the initial movement of each trajectory is along the diagonal *p* = *q*.

**Figure S10.** **Allele frequency trajectories when there are costs to the Rescue allele, but no costs to the Killer allele.** Rescue costs are given by *c*_R2_ = *c*_R1_ = 0.1. There is a stable equilibrium at which both Killer and Rescue are absent, shown by the filled magenta circle. The “Rescue fixed” line of equilibria is shown in green. A trajectory starting at any of the initial conditions either approaches the “both K and R absent” equilibrium or the “Rescue fixed” line of equilibria. Trajectories are colored to indicate their eventual fate. The position of the threshold that separates these two types of trajectories depends on the value of the cost. For higher costs, the threshold moves further away from the “both K and R absent” equilibrium. As the cost approaches zero, the threshold moves towards the value seen in the earlier no cost case.

**Figure S11. Allele frequency trajectories when there are costs to the Rescue allele, with *c*_R2_ > *c*_R1_, but no costs to the Killer allele.** Rescue costs are given by *c*_R1_ = 0, *c*_R2_ = 0.1. There is a stable equilibrium at which both Killer and Rescue are absent, shown by the filled magenta circle. There is an unstable fixed point at which K is fixed but the frequency of R lies between 0 and 1, shown as an unfilled black circle. The “Rescue fixed” line of equilibria is shown in green; this line is repelling. Except for the initial condition with K and R both fixed, trajectories starting at all other initial conditions approach the equilibrium at which both K and R are absent. These trajectories are seen to approach a curve as they move towards this equilibrium; this curve appears to originate at the unstable equilibrium.

**Figure S12. Allele frequency trajectories when there are costs to the Killer allele but not to the Rescue allele.** *c*_K1_ = 0, *c*_K2_ = 0.05, *c*_R1_ = *c*_R2_ = 0. There is an unstable equilibrium at which both K and R are fixed (open green circle) and an attracting “K absent” line of equilibria. All trajectories that start away from the unstable equilibrium approach the “K absent” line.

**Figure S13. Allele frequency trajectories when both killer and rescue alleles have a cost.** Parameter values are *c*_R1_ = 0.2, *c*_R2_ = 0.2, *c*_K1_ = 0.005, and *c*_K2_ = 0.005. The “both K and R fixed” equilibrium (open black circle) is unstable, as is the “R fixed and K absent” equilibrium (open green circle). The “both K and R absent” equilibrium (filled magenta circle) is stable. Trajectories from all initial conditions (except the “both K and R fixed” equilibrium) approach the “both K and R absent” equilibrium: both killer and rescue alleles are lost. As noted earlier, even though the ultimate fate is loss of both K and R, there can be a long transient period in which the rescue allele is present at a high frequency.

VII. **Introduction Threshold Analysis for the No Fitness Costs Case and the Dominant Rescue-Only Cost Case**

When there is no fitness cost to either K or R, we notice that many of the trajectories in Figure S9 are close to being linear. This observation provides a way to obtain an approximate value for the threshold introduction level whose resulting trajectory separates the (*q*, *p*) plane into regions in which trajectories have qualitatively different outcomes. By examining the allele frequencies over the first two generations, we can calculate the direction in which a trajectory leaves the diagonal line *p* = *q*. If the introduced fraction is *f*, then, at the initial time (generation zero) we have that *p* = *q* = *f* and *D* = *f* (1 ‑ *f* ). In the first generation, use of the iterative equations shows that we have *p'* = *q'* = *f* and *D'* = *f* (1 ‑ *f* )/2: in this generation, the allele frequencies don’t change (because all individuals are homozygotes there is no killing of offspring and hence no selection), although the linkage disequilibrium does.

A second application of the iterative equations gives the F2 allele frequencies as

$\begin{aligned} p^{''}=\frac{4f}{\left( f-2 \right)\left( f^{3}-4f^{2}+f-2 \right)} \\ q^{''}=\frac{2f\left( 1+2f-f^{2} \right)}{\left( f-2 \right)\left( f^{3}-4f^{2}+f-2 \right)}\text{.} \end{aligned}$	(6)

The slope of the trajectory leaving the initial point can then be calculated as

$\frac{p^{''}-p}{q^{''}-q}=\frac{f\left( f-4 \right)}{f^{2}-4f+2}$.	(7)

In order for a straight line with this slope to leave the point (*f*, *f* ) and arrive at the corner (0,1), it must have slope (1- *f* )/(-*f*) and so we have

$\frac{1-f}{-f}=\frac{f\left( f-4 \right)}{f^{2}-4f+2}\text{.}$	(8)

Rearranging gives *f* to be the solution of the quadratic

$f^{2}-6f+2=0\text{.}$	(9)

This equation has roots 3±√7, and, since *f* is between 0 and 1, the relevant solution is *f* = 3 –√7 ≈ 0.354, which is a slight overestimate of the value of 0.350 calculated by numerical simulation of the model.

When there are costs to the rescue construct alone and the rescue cost is dominant, we again have an introduction threshold that separates two qualitatively different outcomes (R fixed vs. both K and R lost). When the fitness cost is not close to zero (as in Figure S10), we see that the trajectories in the vicinity of the threshold are far from linear. This prevents us from using the earlier straight-line description to find the approximate value of the introduction threshold. When the fitness cost is small, however, the trajectories in the vicinity of the threshold can again be reasonably approximated by straight lines (see Figure S14). The first motion of the trajectory is, as discussed before, along the diagonal line *p* = *q*, from the point (*p* , *q*) = (*f* , *f* ) to (*p' , q'* ) = (*f ' , f '* ), where

$f^{'}=\frac{f\left( 1-r \right)}{1-2\text{rf}+\text{rf}^{2}}\text{.}$	(10)

A straightforward calculation gives the direction in which trajectories move between the first and second generation as

$\frac{p^{''}-p^{'}}{q^{''}-q^{'}}=\frac{\left( 1+2r-3r^{2}+4r^{3} \right)f^{2}-4\left( 1-r+2r^{2} \right)f+4r}{2r^{2}\left( r-1 \right)f^{3}+\left( 1+4r-r^{2} \right)f^{2}-2\left( 2+r+r^{2} \right)f+2\left( 1+r \right)}\text{.}$	(11)

Here *f* is the introduction fraction and *c*_R2_ = *c*_R1_ = *r*. Assuming that the trajectory continues to move in a straight line with this direction, it will hit the corner at (0,1) if the slope equals (1- *f '*)/(-*f ')*. This leads to the following equation for *f*

$2r^{2}\left( r-1 \right)f^{3}+\left( 1+6r-5r^{2}+2r^{3} \right)f^{2}-2\left( 3-r+2r^{2} \right)f+2\left( 1+r \right)=0\text{.}$	(12)

Figure S15 compares introduction thresholds obtained from (12) to numerical estimates obtained from model simulation over a range of values for the fitness cost *r*. The approximation gives a fair indication of the threshold value, although it underestimates the extent to which its value increases as Rescue becomes increasingly costly.

**Figure S14.** **Allele frequency trajectories when there is a small dominant Rescue cost, but no cost to the Killer allele.** Rescue costs are given by *c*_R2_ = *c*_R1_ = 0.01. Trajectories that are close to the introduction threshold approach the corner at (0,1) along nearly straight-line paths (although trajectories that start just below the threshold curve away at the last moment as they near the corner). This allows an approximate calculation of the introduction threshold, as discussed in the text.

**Figure S15. Introduction threshold for the fixation of the Rescue allele in the dominant Rescue cost, no Killer cost case, as a function of the Rescue cost, *r* = c_R1_ = *c*_R2_.** Values estimated from model simulation are shown as circles, while the approximation obtained from equation (12) is shown as the dashed line.

**Reference:**

Hartl, D.L. and A.G. Clark, 1997. Principles of Population Genetics. Sinauer Associates,Inc. Sunderland, Massachusetts.
